# Spatiotemporal trends in the southwest monsoon wind-driven upwelling in the southwestern part of the South China Sea

**DOI:** 10.1371/journal.pone.0171979

**Published:** 2017-02-10

**Authors:** Poh Heng Kok, Mohd Fadzil Mohd Akhir, Fredolin Tangang, Mohd Lokman Husain

**Affiliations:** 1 Institute of Oceanography and Environment, Universiti Malaysia Terengganu, Kuala Terengganu, Terengganu, Malaysia; 2 School of Environmental and Natural Resource Sciences, Faculty of Science and Technology, Universiti Kebangsaan Malaysia, Bangi, Selangor, Malaysia; 3 School of Marine and Environmental Sciences, Universiti Malaysia Terengganu, Kuala Terengganu, Terengganu, Malaysia; University of Vigo, SPAIN

## Abstract

This study analyzes two wind-induced upwelling mechanisms, namely, Ekman transport and Ekman pumping that occur during the southwest monsoon. The results suggest that the coastline of the east coast of Peninsular Malaysia (ECPM) is affected by upwelling with spatiotemporal variations. Characterization of upwelling by using wind-induced upwelling indexes (*UI*_*W*_) indicate the existence of favorable upwelling conditions from May to September. Upwelling intensity increased in May and peaked in August before declining in September, decreasing intensity from the southern tip towards the northern tip along the coastline of the ECPM. The existence of upwelling along the ECPM has resulted in an important difference between the SSTs of the inshore and the oceanic regions. Nonetheless, the use of the SST gradient between the inshore and the oceanic SSTs to characterize upwelling (*UI*_*SST*_) was found to be unsuitable because the SST along the ECPM was affected by water advection from the Java Sea and incessant changes in the SST. In order to indicate the major contributor of wind-induced upwelling along the ECPM in terms of the spatiotemporal scale, a comparison between Ekman transport and Ekman pumping was drawn by integrating Ekman pumping with respect to the distance where the positive wind stress curl existed. The estimation of Ekman transport and Ekman pumping indicated that Ekman pumping played a major role in contributing towards upwelling in any particular month during the southwest monsoon along the entire coastline of the ECPM as compared to Ekman transport, which contributed towards more than half of the total upwelling transport. By dividing the ECPM into three coastal sections, we observed that Ekman pumping was relatively predominant in the middle and northern coasts, whereas both Ekman transport and Ekman pumping were equally prevalent in the southern coast.

## Introduction

Upwelling has been traditionally described as a process that significantly affects the primary productivity and fishery production because it involves the wind-driven motion of cooler and nutrient-rich water from the deeper ocean layers to the surface, thereby replacing the nutrient-depleted surface water [1,2]. Wind-driven upwelling can be classified into two processes, i.e., Ekman transport and Ekman pumping [3–5]. In the case of Ekman transport, the alongshore prevailing winds exert a uniform Coriolis force to drive the surface water either offshore or onshore, which in turn pushes the deeper coastal water upward or downward. In the case of Ekman pumping, positive wind stress curl engenders ocean surface divergence, forcing the upward movement of water, resulting in Ekman suction, which in turn causes upwelling, while negative wind stress curl induces surface convergence, forcing downward movement of water, resulting in Ekman pumping, which in turn causes downwelling [6–8]. For the sake of convenience, Ekman suction will be referred to as Ekman pumping.

The Ekman transport component perpendicular to the coastline and Ekman pumping have been commonly used for characterizing upwelling (hereafter referred to as the wind upwelling index, *UI*_*W*_). In addition to Ekman transport and Ekman pumping, some authors have considered the sea surface temperature (SST) gradient between the coastal water and oceanic water as an alternative parameter to characterize upwelling (hereafter referred to as the SST upwelling index, *UI*_*SST*_) [9–14]. Nonetheless, *UI*_*W*_ and *UI*_*SST*_ are not used without their caveats. The disadvantage of using *UI*_*W*_ is that it only refers to the potential effect of winds on the sea surface and does not represent the actual oceanic variable. With regard to *UI*_*SST*_, the temperature differences between the coastal water and the oceanic water cannot be directly attributed to upwelling because the water temperature is easily affected by local and large-scale external factors, such as freshwater input, large-scale atmospheric phenomena, and synoptic-scale weather and climate systems [9,12].

Despite the fact that the wind-driven upwelling processes of Ekman transport and Ekman pumping have been proved to induce upwelling, their contribution differ. Near Cabo Frio, Brazil, Ekman pumping was more predominant than Ekman transport [7], whereas in the Yucatan Peninsula, Ekman transport appeared to be more significant than Ekman pumping [8]. On the other hand, in the California Current System, Ekman transport is as significant as Ekman pumping [6].

**Fig 1:**
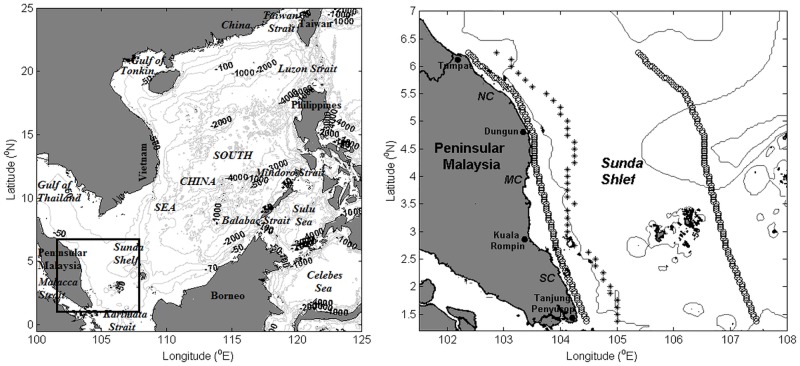
Map and bathymetry of the SCS and the ECPM. Isobaths of 50 m, 70 m, 100 m, 1000 m, 2000 m, 3000 m, and 4000 m are presented. The rectangle on the left panel denotes the location of the ECPM. On the right panel, the asterisk points represent the latitudinal transect where Ekman transport and Ekman pumping are considered and the black circles represent the latitudinal transects where the SST data are obtained. SC denotes the southern coast (Tanjung Penyusop to Kuala Rompin); MC, the middle coast (Kuala Rompin to Dungun); and NC, the northern coast (Dungun to Tumpat). The shoreline and bathymetry data are obtained from Global Self-consistent, Hierarchical, High-resolution Geography Database (GSHHG) [15] and 2-minute Gridded Global Relief Data (ETOPO2v2) [16], respectively.

The east coast of Peninsular Malaysia (ECPM) is located in the southwestern part of the South China Sea (SCS). The SCS, listed as the largest semi-enclosed marginal sea in the western Pacific Ocean, extends from the equator to 25°N and from 99–122°E. It is surrounded by China, Vietnam, Peninsular Malaysia, Borneo, the Philippines, and Taiwan; and is connected to the East China Sea, Indian Ocean, Java Sea, Sulu Sea, and the Pacific Ocean through several adjacent straits. The average depth of the SCS is approximately 2000 m, with the maximum depth exceeding 4000 m in the center basin (Fig 1).

The SCS is dominated by the East Asian Monsoon [17,18]. In boreal winter (northeast monsoon), the northeasterly wind prevails in the SCS from November to March, whereas in boreal summer (southwest monsoon), the direction of the wind is reversed to southwesterly, and it prevails from May to September. On the other hand, the period between the northeast and southwest monsoon is dominated by the inter-monsoon, where the wind prevails in a variable direction, and it usually occurs in April and October [19,20]. During the southwest monsoon, southwesterly-prevailing winds with the north-south orientation of the coastline provide wind-induced upwelling conditions along the west coast of the SCS [18,21–24]. Nonetheless, the existing studies on upwelling along the ECPM are relatively new, fragmented, and rather limited.

Upwelling was initially proven to exist along the ECPM during the southwest monsoon, where a classical upwelling sign of the onshore shoaling of thermohaline towards the coast and the surface was observed, forming an elongated cooler SST along the coast [25]. The argument presented in Akhir et al. [25] and Daryabor et al. [26] based on the theoretical definition suggested that the alongshore prevailing winds during the southwest monsoon induced offshore Ekman transport, which caused upwelling. Besides, Kok et al. [27] added that the positive wind stress curl observed during the southwest monsoon induced upwelling along the ECPM.

Understanding of this upwelling characteristic in the region is relatively new, and related studies have yet to ascertain details on the dynamics of the upwelling system during the southwest monsoon upwelling along the ECPM. Therefore, numerous questions have not yet been answered. For example, what are the temporal and spatial scales of upwelling? Which upwelling index is more appropriate for characterizing upwelling? Which wind-driven upwelling mechanism is the major contributor of upwelling? The answers to these questions are vital for the food and environment securities that will assist in developing a better strategy for managing and conserving the fishery resources and environment. The key objectives of this study are to provide the dynamical characteristics of the upwelling system, which include examination of the spatiotemporal variability of wind-driven upwelling and to characterize upwelling along the ECPM in terms of *UI*_*W*_ and *UI*_*SST*_. In addition, the objective of this study is to compare both wind-driven upwelling mechanisms and to determine the major contributor of upwelling in terms of a spatiotemporal scale along the ECPM during the southwest monsoon.

## Data and methods

### Wind data and *UI*_*W*_

The 10-m zonal and meridional wind components with a resolution of 0.125° × 0.125° were obtained from the European Centre Medium-range Weather Forecast (ECMWF) Interim Reanalysis (ERA-Interim) on a monthly basis from 2003 to 2013. ERA-Interim is a global atmospheric reanalysis product that combines model information and observations of several different sources in an optimum manner to create a consistent, global, and an optimum estimate of various atmospheric and oceanographic parameters. Comprehensive documentation of the ERA-Interim parameters was provided by Berrisford et al. [28]. ERA-Interim used the 2006 version of the Integrated Forecast System (IFS) (Cy31r2) for data assimilation that encompasses a four-dimensional variation analysis (4D-Var) with a 12-h analysis window. Further details on the assimilation system used in the ERA-Interim can be found from the IFS Cy31r1 documentation [29] and the configuration and accomplishment of the ERA-Interim data assimilation system were provided by Dee et al. [30]. The monthly data obtained from the ERA-Interim were averaged over the same month from May to September during 2003–2013 to represent the monthly climatology mean during the southwest monsoon.

#### Ekman transport and *UI*_*ET*_

Ekman transport (*m*^3^ *S*^−1^ *m*^−1^) at each grid point was calculated on the basis of the wind products obtained from the ERA-Interim by using the following equation:

$$Q_{x}=\frac{\tau_{y}}{\rho_{w}f}$$

$$Q_{y}=-\frac{\tau_{x}}{\rho_{w}f}$$ (1)

Where *τ* is the wind stress, *ρ*_*w*_ = 1025 *kg m*^−3^ is the density of seawater, and *f* = 2*Ωsinθ* is the Coriolis parameter (*Ω* = 7.292 × 10^−5^ *rad s*^−1^ is the Earth’s angular velocity, and *θ* is the latitude). The *x* and *y* subscripts refer to the zonal and meridional wind components of Ekman transport, respectively. *τ* (*N m*^-2^) is calculated as follows:

$$\tau_{x}=\;\rho_{a}C_{d}{\left(W_{x}{}^{2}+W_{y}{}^{2}\right)}^{\frac{1}{2}}W_{x}$$

$$\tau_{y}=\rho_{a}C_{d}{\left(W_{x}{}^{2}+W_{y}{}^{2}\right)}^{1/2}W_{y}$$ (2)

where *ρ*_*a*_ = 1.22 *kg m*^−3^ is the density of air, *C*_*d*_ = 1.3 × 10^−3^ is the constant dimensionless drag coefficient, and *W* is the wind speed at 10 m. However, a higher drag coefficient of *C*_*d*_ = 2.6 × 10^−3^ was used in this study to roughly compensate for the underestimation of the wind stress caused by using mean wind data in the above nonlinear equations, as described by Bakun [31], Bakun & Nelson [32] and Trenberth et al. [33]. This higher value of dimensionless drag coefficient was also adopted by Kok et al. [27] to calculate the monthly wind stress along the ECPM.

In order to characterize upwelling by using Ekman transport, the calculated Ekman transport was resolved to the transport perpendicular to the coastline, i.e., *UI*_*ET*_, by the mean of

$$UI_{ET}=-\left(\text{sin}\left(\varphi -\frac{\pi }{2}\right)Q_{y}+\text{cos}\left(\varphi -\frac{\pi }{2}\right)Q_{x}\right)$$ (3)

where *φ* is the angle between the coastline and the equator. The coastline of the ECPM is highly irregular on a relatively small scale; however, the coastline angle shows a significant change in the major scale, where it is 45° from Tumpat to Dungun (northern coast), 90° from Dungun to Kuala Rompin (middle coast), and 66° from Kuala Rompin to Tanjung Penyusop (southern coast). Hence, *φ* was substituted according to the coastline angle at each section of the coast. On the basis of the definition of *UI*_*ET*_, a positive value corresponds to a favorable upwelling condition, whereas a negative value corresponds to an unfavorable upwelling condition.

#### Ekman pumping and *UI*_*EP*_

In the case of Ekman pumping, *UI*_*EP*_ (*m s*^−1^) of each grid point was calculated by using the following equation:

$$UI_{EP}=\frac{curl\;\left(\tau \right)}{\rho_{w}f}$$ (4)

where *curl* (*τ*) is the wind stress curl (*N m*^−3^), which is calculated as follows:

$$curl\;\left(\tau \right)=\frac{\partial \tau_{y}}{\partial_{x}}-\frac{\partial \tau_{x}}{\partial_{y}}$$ (5)

Similar to the case of *UI*_*ET*_, a positive value of *UI*_*EP*_ indicates a favorable upwelling condition, whereas a negative value of *UI*_*EP*_ indicates an unfavorable upwelling condition.

### Sea surface temperature and *UI*_*SST*_

The SST was obtained from the Moderate-resolution Imaging Spectrometers (MODIS) onboard the Terra and Aqua satellites. Data can be assessed from the Ocean Color Web [34]. The Terra and Aqua satellites were launched in December 1999 and May 2002, respectively. Terra was set to orbit the Earth in the morning from north to south over the equator. Conversely, Aqua was set to orbit the Earth from south to north across the equator in the afternoon. Terra and Aqua take approximately 1–2 days for viewing the entire Earth’s surface, acquiring multidisciplinary data in 36 spectral bands, wavelengths, or groups.

In the present study, level 3, 4-km resolution, 4-μ nighttime SSTs acquired from both the Terra and the Aqua satellites were combined and then averaged over the same month from May to September during 2003–2013 to represent the monthly SST climatology during the southwest monsoon.

With the objective of characterizing upwelling by using SST (*UI*_*SST*_), the SST gradient between the coastal (*SST*_*Coast*_) and the oceanic (*SST*_*Ocean*_) temperatures was determined by taking the mean of

$$UI_{SST}=SST_{Coast}-SST_{Ocean}$$ (6)

where *SST*_*Coast*_ is the SST nearest to the coast, and *SST*_*Ocean*_ is the SST along the same latitude that is 3° further east (Fig 1). Therefore, a negative *UI*_*SST*_ value implies that the coast is cooler than the oceanic temperature, indicating a favorable upwelling condition, whereas a positive *UI*_*SST*_ value implies that the coast is warmer than the oceanic temperature, indicating a unfavorable upwelling condition. *SST*_*Coast*_ was set slightly away from the coast near 3.5°N because this region is altered by a significant river runoff, which will lead to the generation of inaccurate results.

### Comparison of wind-driven upwelling processes

For the comparison and estimation of the two wind-driven upwelling processes, i.e., Ekman transport and Ekman pumping, the method proposed in Pickett & Paduan [6] was adopted. A direct comparison between Ekman transport and Ekman pumping was drawn. First, Ekman transport that is perpendicular to the coastline located at each grid point nearest to the ECPM’s coastline was determined. Second, Ekman pumping was converted to vertical transport (*m*^3^ *s*^−1^ *m*^−1^) by integrating Ekman pumping from the grid points, nearest to the coast up to the distance where the wind stress curl remained positive along the direction perpendicular to the coastline. The distance along which the wind stress curl remained positive was estimated by referring to the previous publication by Kok et al. [27]. Therefore, once Ekman pumping was transformed to vertical transport, two independent upwelling estimates were available, i.e., upwelling due to Ekman transport and that due to integrated Ekman pumping, enabling the comparison between these two upwelling mechanisms.

## Results

**Fig 2:**
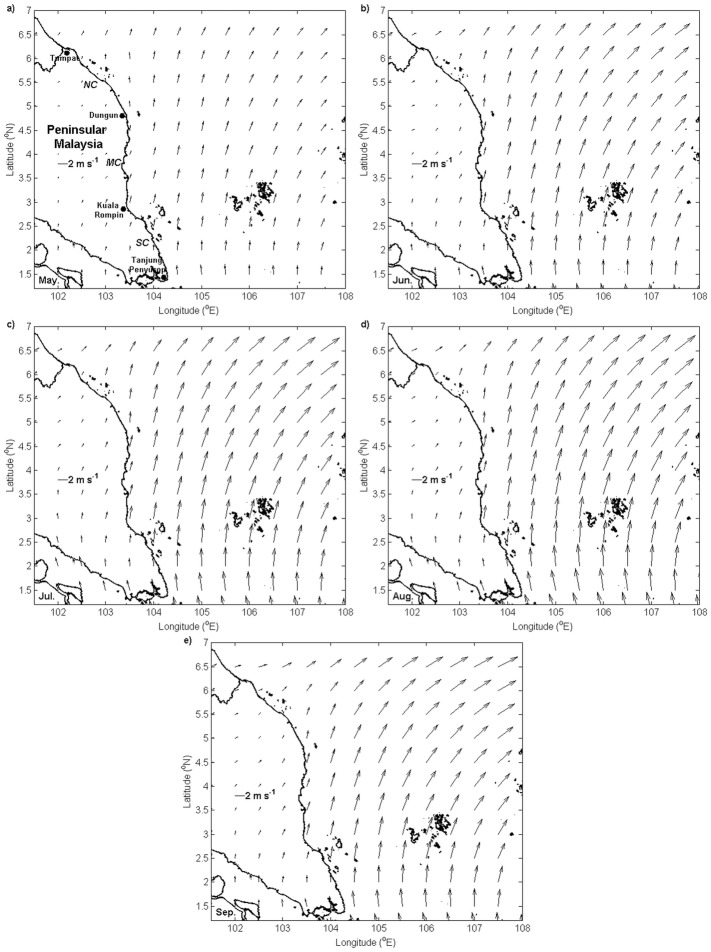
Monthly climatology of surface wind vectors and magnitude (unit: m s^-1^). (a) May, (b) June, (c) July, (d) August, and (e) September.

During the southwest monsoon, the south to southwesterly winds were dominated by the meridional component that prevailed over the southwestern part of the SCS; these winds ran alongshore, roughly parallel to the coastline along the southern and middle coasts, and almost in a perpendicular direction, which was directed offshore along the northern coast of the ECPM. Apart from its orientation, the magnitude of the winds also showed an important variation. In general, the wind speed gradually increased in May and peaked in August before declining in September. Furthermore, during these months, the wind speed was recorded to be the highest in Tanjung Penyusop, and it decreased as it moved towards Tumpat; further, the wind speed was found to increase seaward up to a certain distance as it deviated from the coastline of the ECPM during the southwest monsoon, except the region from 5.9°N until Tumpat (Fig 2).

### Ekman transport and *UI*_*ET*_

**Fig 3:**
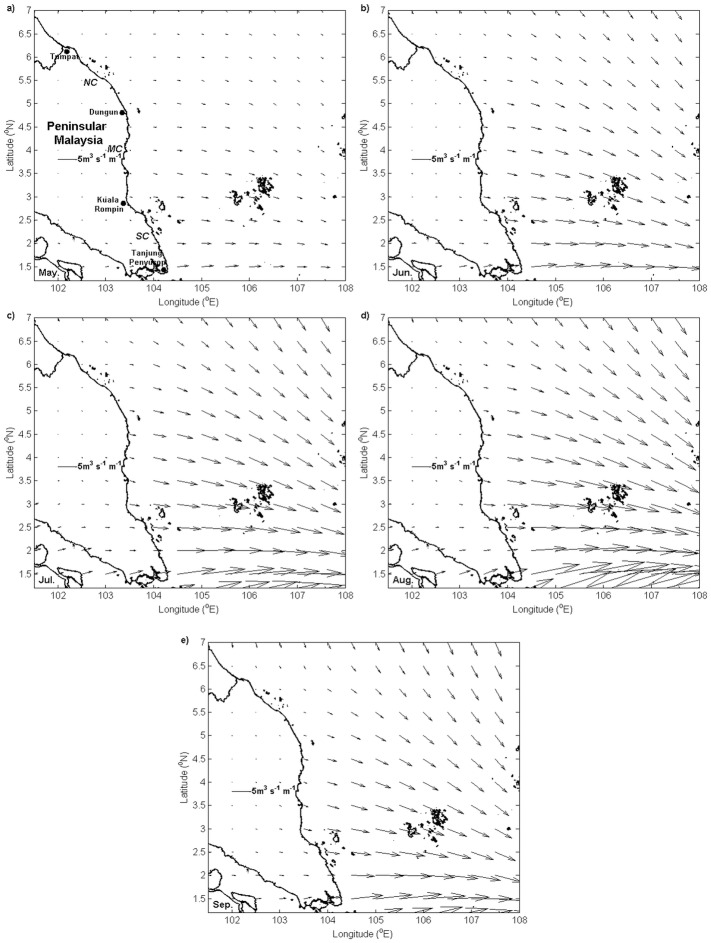
Ekman transport (unit: m^3^ s^-1^ m^-1^) monthly climatology. (a) May, (b) June, (c) July, (d) August, and (e) September.

Fig 3 shows the Ekman transport monthly climatology pattern along the ECPM computed from the ECMWF wind field products. Throughout the southwest monsoon, several characteristics of Ekman transport remain unchanged, where the Ekman transport was mostly meridional and directed eastward perpendicular to the coastline in the offshore direction along the southern and middle coasts, whereas in the northern coast, the Ekman transport was directed southeastward, running roughly parallel to the coast. Despite the quasi-constant orientation, the intensity of Ekman transport was observed to be modulated on a monthly basis during the southwest monsoon. In particular, the intensity of Ekman transport increased from May to August before declining in September. Moreover, the intensity of Ekman transport during the southwest monsoon months declined as it moved northward and it increased as it moved seaward over some distances.

**Fig 4:**
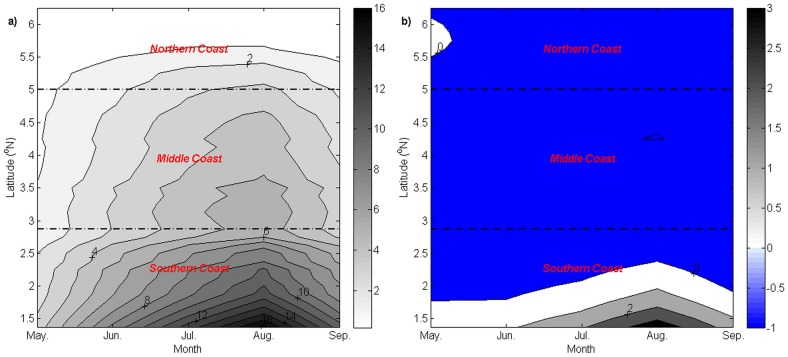
Monthly climatology of latitudinal Ekman transport (unit: m^3^ s^-1^ m^-1^) during the southwest monsoon. (a) *Q_x_* and *(b) Q_y_*.

Fig 4a and 4b show the monthly mean latitudinal zonal (*Q*_*x*_) and meridional (*Q*_*y*_) components of the Ekman transport climatology from 2003 to 2013 during the southwest monsoon along the latitudinal transect, as shown in Fig 1. The obtained results indicated that *Q*_*y*_ did not show much variation and exhibited a smaller value than *Q*_*x*_. A positive value of *Q*_*x*_ (eastward zonal component) was observed from May to September, where the intensity gradually increased in May and peaked in August before declining in September; the highest intensity was observed in Tanjung Penyusop, which decreased towards Tumpat. Further, it was recorded that a relatively large part of the coast was dominated by a constant negative value (southward meridional component) of *Q*_*y*_ in any particular month during the southwest monsoon, and only a small part in the southern coast was subjected to a positive value (northward meridional component) of *Q*_*y*_; this positive value exhibited a litter variation and it increased from June and peaked in August before decreasing in September.

Conventionally, maximum upwelling occurs when Ekman transport is perpendicular to the coast. Hence, the determination of *UI*_*ET*_ that resolves the zonal and meridional components of Ekman transport into Ekman transport perpendicular to the coastline is a good estimate to characterize upwelling, particularly the spatial variability of coastal upwelling along the ECPM that exhibits a considerable change in the coastline orientation from Tanjung Penyusop to Tumpat.

**Fig 5:**
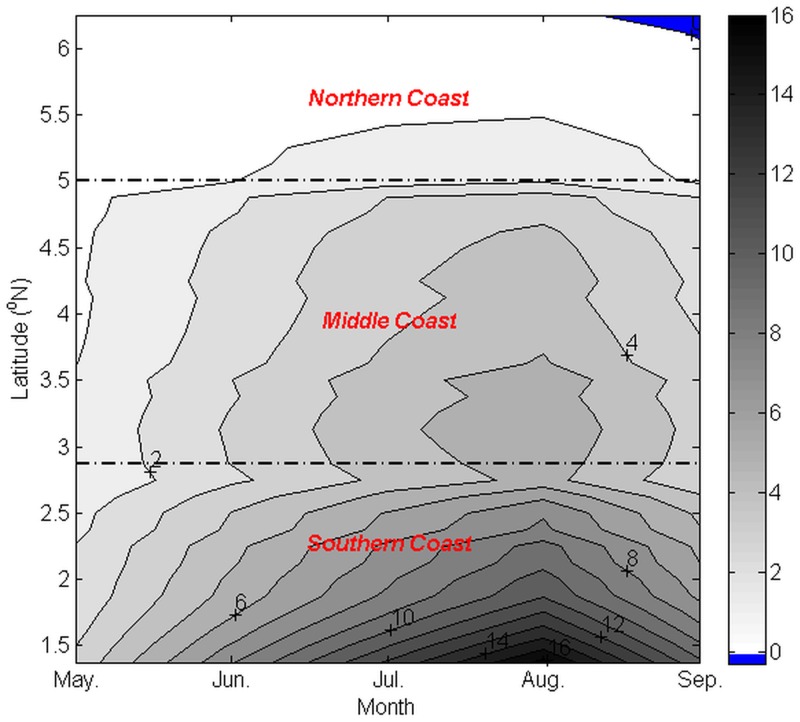
Monthly climatology of latitudinal *UI_ET_* (unit: m^3^ s^-1^ m^-1^) during the southwest monsoon.

Fig 5 shows the monthly characteristics of *UI*_*ET*_ throughout the latitudinal transect, as shown in Fig 1, deduced from the monthly climatology data collected over 11 years. The obtained *UI*_*ET*_ was similar to *Q*_*x*,_ where the positive value of *UI*_*ET*_ steadily increased from May and peaked in August before declining in September. Furthermore, the intensity of *UI*_*ET*_ was the highest in Tanjung Penyusop, and it gradually reduced as it travelled towards the northern region.

### Ekman pumping and *UI*_*EP*_

**Fig 6:**
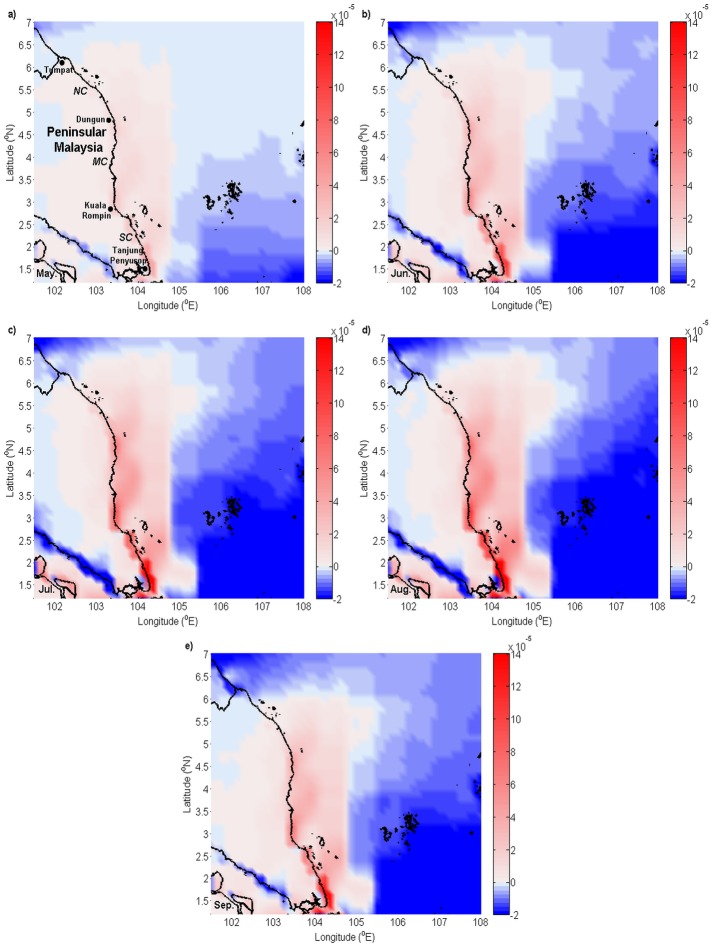
Ekman pumping (unit: m s^-1^) monthly climatology. (a) May, (b) June, (c) July, (d) August, and (e) September.

Fig 6 shows that Ekman pumping along the ECPM was positive from Tanjung Penyusop to 5.9°N; however, Ekman pumping was reported to be negative from 5.9°N to Tumpat in the northern coast during the southwest monsoon months. Moreover, the Ekman pumping intensity was perceived to exhibit a spatial change along the coastline of the ECPM where the intensity of Ekman pumping was the highest in Tanjung Penyusop and it decreased northward. In addition to the spatial variability, Ekman pumping also exhibited important temporal variability, where the intensity of Ekman pumping increased from May and peaked in August before declining in September.

### Sea surface temperature and *UI*_*SST*_

**Fig 7:**
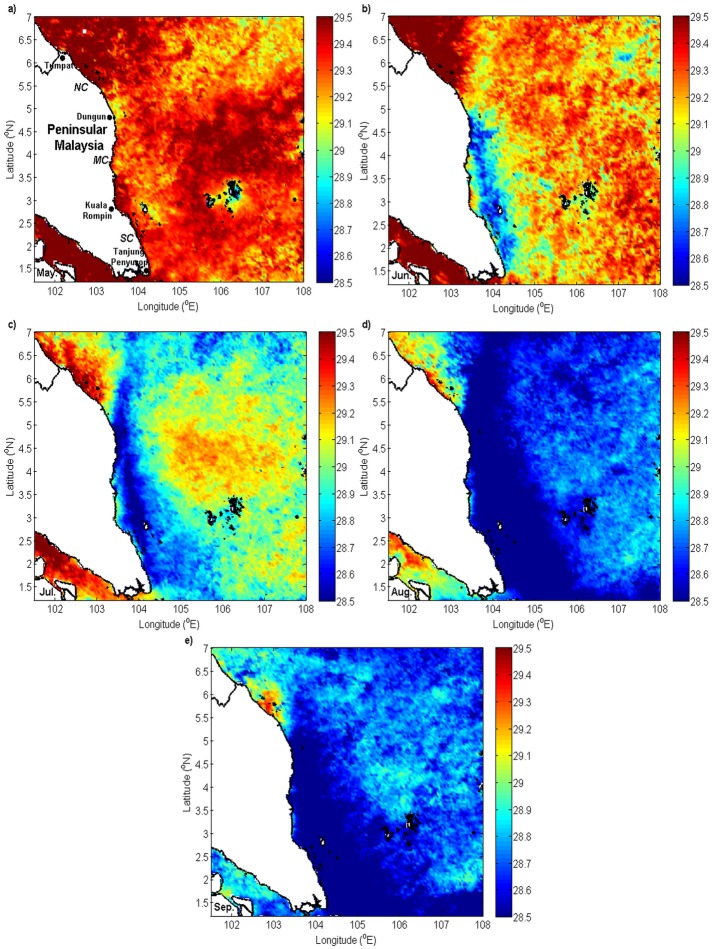
Monthly climatology of SST (unit: °C). (a) May, (b) June, (c) July, (d) August, and (e) September.

The monthly temporal and spatial variability of the SST from 2003 to 2013 during the southwest monsoon along the ECPM is shown in Fig 7. As observed from the SST climatology map, a relatively clear patch of cooler water was observed in the inshore region from June to September. In June, cooler water was found to be present in the southern and middle coasts; nevertheless, the cooler water from July to September was found to be extended from the southern region of the ECPM, with an increasing width from July to August before decreasing in September. The SST along this patch of cooler water decreased from June to August and remained similar in September. There was a fascinating feature occurred in the inshore region of July where a distinguishable cooler water was bounded by the slightly warmer water that stretched from the southern region.

In addition to the fluctuation of temperature along the coast due to the presence of cooler water, the oceanic temperature also experienced changes, where the oceanic SST gradually decreased from May to August and slightly increased from August to September. The direct comparison between inshore and offshore SST signposted that the SST in the inshore region was slightly cooler than the offshore region, reflected the existence of the upwelling conditions, particularly in June; however, this was not the case from July to September, where the patch of cooler water was transformed into a larger band and stretched from the south of the ECPM, indicating that another source of cooler water was transported into the ECPM.

The difference with the other sections of the coast was that, in the northern coast of the ECPM, the SST was warmer in the inshore region and the cooler water was observed to be located slightly away from the coast and extended northward from the southern region. Moreover, a warmer SST was also observed in the region centered at 3.5°N in the middle coast, which it can be differentiated from the cooler water patch.

**Fig 8:**
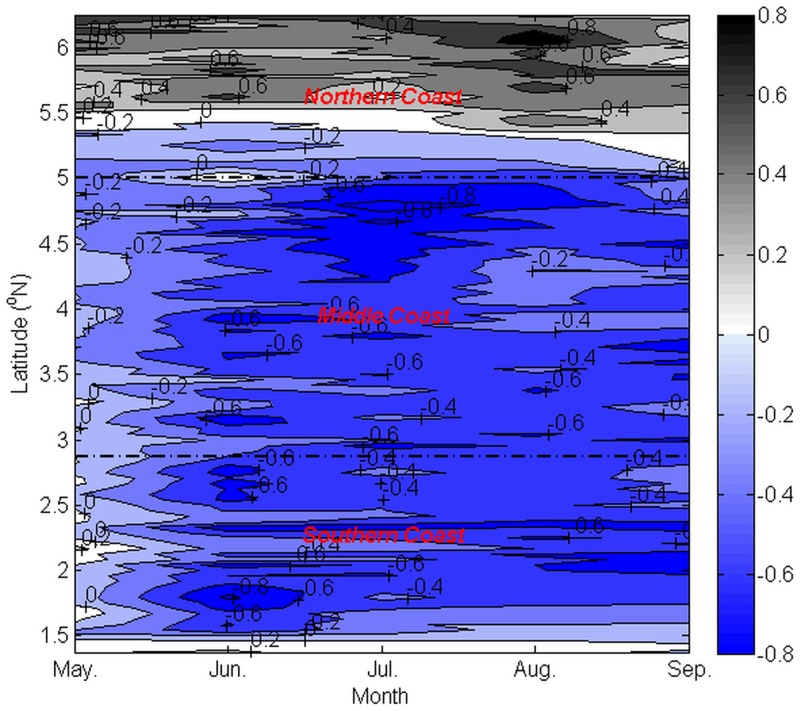
Monthly climatology of latitudinal *UI_SST_* (unit: °C) during the southwest monsoon.

The formation of a cooler water patch in the inshore region created a temperature difference between the inshore and the offshore regions. The computed monthly latitudinal *UI*_*SST*_ climatology during the southwest monsoon from 2003 to 2013 presented a monthly alongshore SST gradient along the ECPM (Fig 8).

A negative SST gradient was observed along the southern and middle coasts and the south of 5.5°N in the northern coast during the southwest monsoon. Comparatively, a higher negative temperature gradient of -0.44–0.50°C was observed from June to September along the southern and middle coasts.

### Comparison between Ekman transport and Ekman pumping

In order to quantify the relative significance of the monthly wind-driven coastal upwelling of Ekman transport and Ekman pumping, the estimates of Ekman transport and Ekman pumping were integrated along the coastline of the ECPM (Table 1). Nonetheless, from the Section 3.2, it was perceived that Ekman pumping was only favorable for upwelling until 5.9°N in the northern coast. In order to make the comparison of Ekman transport and Ekman pumping became possible, hence, the integration of Ekman transport and Ekman pumping estimates were only considered from Tanjung Penyusop to 5.9°N in the northern coast. The total transport was estimated by summing up Ekman transport and Ekman pumping.

**Table 1:** Southwest monsoon monthly upwelling estimates and their percentage contribution. The estimations were based on 11 years’ average, from 2003 to 2013, integrated along the coastline of the ECPM.

Month	Upwelling Transport (m^3^ s^-1^ m^-1^)			Percentage Contribution (%)	
	Ekman Pumping	Ekman Transport	Total Transport	Ekman Pumping	Ekman Transport
May	1.01	0.64	1.65	61.28	38.72
June	2.29	1.57	3.86	59.25	40.75
July	3.41	2.41	5.82	58.63	41.37
August	4.55	2.94	7.49	60.78	39.22
September	2.66	1.84	4.50	59.14	40.86

The estimations of Ekman transport and Ekman pumping showed significant monthly variability. In May, both Ekman transport and Ekman pumping were low; however; Ekman pumping was 22.56% stronger than Ekman transport. Further, both Ekman transport and Ekman pumping increased and attained their maximum magnitudes in August, where Ekman pumping was still predominant over Ekman transport in the range between 59.25% and 60.78%. Nonetheless, in September, both the upwelling transport mechanisms decreased, and Ekman transport was recorded to be 18.28% weaker than Ekman pumping. Estimation of the total transport exhibited a similar trend for both the transport mechanisms as their magnitudes increased in May and peaked in August before declining in September.

**Fig 9:**
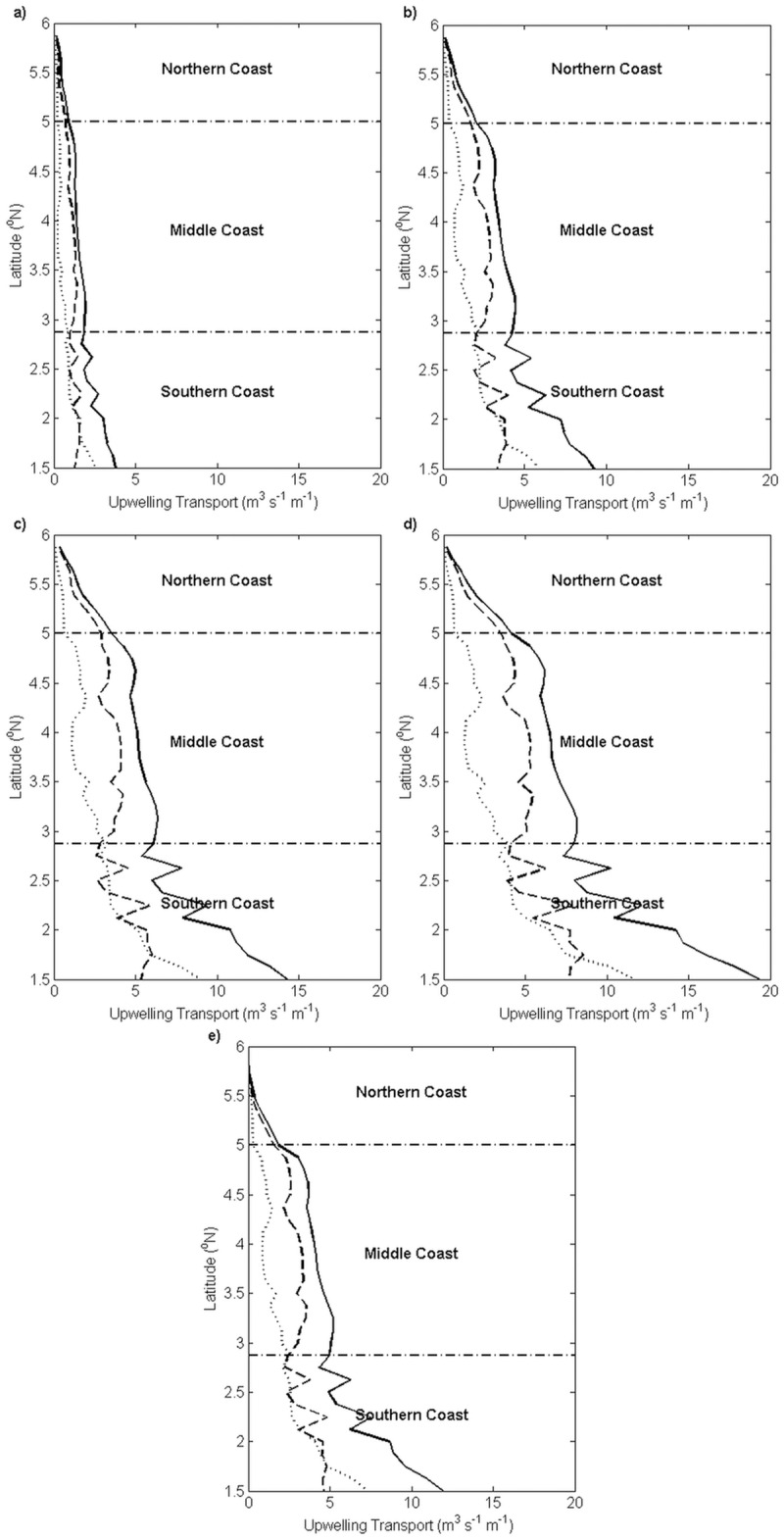
Monthly climatology of alongshore wind-induced upwelling estimates (unit: m^3^ s^-1^ m^-1^) along the ECPM. (a) May, (b) June, (c) July, (d) August, and (e) September. The estimations were based on 11 years’ average, from 2003 to 2013. The dotted lines represent the Ekman transport, the dashed lines represent the Ekman pumping and the solid lines represent the total upwelling transport.

With the objective of assessing the contribution of upwelling between Ekman transport and Ekman pumping to the total upwelling transport on a spatial scale, Ekman transport and integrated Ekman pumping were plotted for each month during the southwest monsoon along the coastline of the ECPM from Tanjung Penyusop in the southern coast to 5.9°N in the northern coast (Fig 9).

In the case of the southwest monsoon, the spatial trend in Ekman transport, Ekman pumping, and the total transport across May, June, July, August, and September manifested a similar pattern, but with temporal monthly variability, as described.

In general, across three sections of the coast from Tanjung Penyusop to 5.9°N from May to September, the magnitudes of Ekman transport, Ekman pumping, and the total transport diminished. In all the sections of the coast, the magnitude of Ekman pumping was typically greater and more variable than Ekman transport in the southern coast. Ekman transport and Ekman pumping exhibited similar patterns in the middle and northern coasts. Along the coastline of the ECPM, the total transport and Ekman pumping were observed to exhibit a similar trend, except south of 1.7°N in the southern coast where the trend in Ekman pumping was similar to that in Ekman transport.

**Fig 10:**
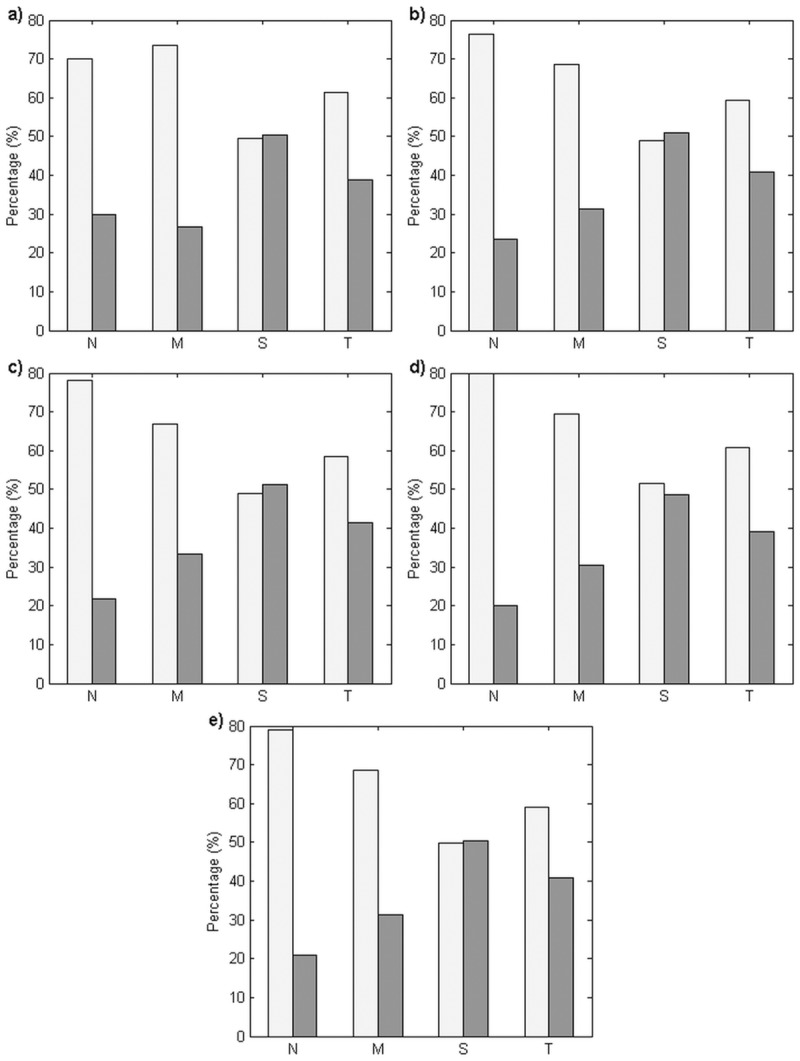
Percentage contribution of Ekman transport and Ekman pumping (unit: %). (a) May, (b) June, (c) July, (d) August, and (e) September. The calculation was based on 11 years’ average, from 2003 to 2013. N, M, and S denote the northern, middle, and southern coasts, respectively, whereas T denotes the entire coastline of the ECPM. The bars in grey and dark grey represent the Ekman pumping and Ekman transport, respectively.

In order to obtain a better insight into the wind-driven upwelling contribution in each section of the coast, the percentage contributions of Ekman transport and Ekman pumping were investigated (Fig 10). It was observed that Ekman transport played a limited role in generating upwelling in the northern and the middle coasts, the percentage contributions of which were only 20.16%–29.97% in the northern coast and 26.57%–33.23% in the middle coast, from May to September. On the other hand, in the southern coast, each of the transport mechanisms shared approximately half of the contribution. Because Ekman pumping was observed to dominate upwelling in two out of the three sections of the coast in each month, it was apparent that Ekman pumping plays a significant role in generating upwelling along the entire coastline of the ECPM, with a percentage contribution of 61.28% in May, 59.25% in June, 58.63% in July, 60.78% in August, and 59.14% in September.

## Discussion

Upwelling is a term that is frequently used to show various conditions comprising the upward movement of water; it regularly causes a misperception in explaining the consequence of upwelling rather than the process itself. In this study, upwelling was investigated through wind-driven upwelling processes and was characterized using the wind (*UI*_*W*_) and the SST upwelling (*UI*_*SST*_) indexes. Through these analyses, we have determined the major contributor of upwelling along the ECPM.

The prevailing winds along the ECPM during the southwest monsoon experienced spatial and temporal variability. In the case of the southwest monsoon, winds prevailed along the north to the northeast direction by the meridional component of winds, with a rising speed from May to August before diminishing in September. These south to southwesterly winds ran parallel along the southern and middle coasts before changing their course to being almost perpendicular to the coastline along the northern coast of the ECPM. Under the influence of Earth’s rotation, the net transport of water was directed 90° to the right of the north or northeast direction of the prevailing winds and caused Ekman transport dominated by its zonal component. This condition resulted in cross-shore Ekman transport in the southern and middle coasts that was favorable for upwelling, whereas Ekman transport in the northern coast ran almost alongshore, which was somehow less favorable for upwelling.

Wind direction is crucial for determining the direction of Ekman transport, whereas the wind speed and it associated wind stress are crucial for determining the intensity of Ekman transport. This was evident from the investigation where Ekman transport increased in May and peaked in August before declining in September as the wind speed changed during the southwest monsoon. In addition, from Eq 1, it is inferred that Ekman transport is inversely proportional to the sine of latitude (i.e., Coriolis parameter); hence lower the latitude, higher is the intensity of Ekman transport [35]. Therefore, the north-south coastline orientation of the ECPM led to an increase in the Ekman transport intensity from a higher to a lower latitude.

It is well known that the areas with north-south orientation of coastline, the major contributor of upwelling is *Q*_*x*_ (across-shore component). However, in this study, *Q*_*y*_ (alongshore component) has also been taken into consideration. Along the ECPM, it was perceived that Ekman transport was dominated by eastward *Q*_*x*_ instead of northward and southward *Q*_*y*_ because *Q*_*x*_ was apparently higher than *Q*_*y*_. The domination of eastward *Q*_*x*_ causes water to move away from the ECPM, which in turn triggers upwelling. Even though the examination of *Q*_*x*_ and *Q*_*y*_ yields an ideal upwelling condition, we cannot characterize upwelling because it does not represent the offshore movement of water that is running orthogonal to the local coastline. Therefore, *UI*_*ET*_ that resolves both the Ekman transport components perpendicular to the coastline is used to determine the offshore component of Ekman transport. Nevertheless, the intensity of Ekman transport as determined using *Q*_*x*_ and *UI*_*ET*_ was similar, and in both cases, the intensity increased in May and peaked in August before declining in September, with an increasing magnitude southward that was probably attributed to the less obvious change in the ECPM’s coastline angle.

Ekman pumping or *UI*_*EP*_ is another *UI*_*W*_ other than *UI*_*ET*_ that is used to characterize upwelling. Along the ECPM, poleward alongshore winds increased seaward over some distances. The seaward increase in the poleward alongshore winds with the coastline orientated to the left of the winds in the Northern Hemisphere is characterized by cyclonic winds, whereas the shoreward increase in the poleward alongshore winds is characterized by anti-cyclonic winds. In the case of the cyclonic winds, the offshore component of Ekman transport increases seaward and generates ocean surface divergence, resulting in Ekman pumping, which is favorable for upwelling [6,7,36]. Thus, the poleward alongshore wind stress that decreased seaward along the ECPM generated cyclonic winds, induced upwelling, and was indicated by a positive value of *UI*_*EP*_ with the intensity increasing in May and peaking in August before declining in September. However, the observation was different in the region from 5.9°N to Tumpat, where the alongshore prevailing wind stress decreased seaward and generated a negative value of *UI*_*EP*_ (anti-cyclonic winds), which was unfavorable for upwelling. As in the case of *UI*_*ET*_, the calculation of *UI*_*EP*_ involved the sine of latitude (Eq 4); hence, *UI*_*EP*_ decreased with an increase in the latitude from south to north.

In general, throughout the southwest monsoon, the oceanic water along the ECPM showed variability in the monthly SST. The composite analysis of the SST in the SCS during the southwest monsoon exhibited variation in the SST, which was affected by the suppression of the surface latent heat flux and the downward short-wave radiation flux [37]. Hence, it was predicted that this variability in the latent heat flux and short-wave radiation flux in the SCS contributed to the monthly variability in the SST, particularly in the oceanic water near the ECPM during the southwest monsoon. Even though the water around the ECPM experienced temperature changes, the formation of inshore water cooler than the offshore water was somehow apparent. During the southwest monsoon, a clearer patch of cooler water in contrast with the warmer offshore water was observed from June to September, forming thermal fronts that extended along the coast, which occupied the southern and middle coasts of the ECPM, indicated extensive upwelling along the ECPM [25–27]. The region centered at 3.5°N in the middle coast is typically influenced by riverine outflow from the Pahang River and caused a river plume with warmer SST in the inshore region, which can be distinguished from the cooler water patch [27,38].

The horizontal contrast in temperature along the latitude was observed through the computed *UI*_*SST*_. From the results of the investigations, a negative *UI*_*SST*_ was observed from May to September along the southern and middle coasts. In May, although a negative *UI*_*SST*_ was observed, this gradient was typically small (<0.4°C) and was unable to form clearer thermal fronts, as indicated by the SST maps. Conversely, clearer thermal fronts were observed from June to September along the southern and middle coasts, with a gradient of more than 0.4°C.

Typically, the value of *UI*_*SST*_ was consistent with the values of *UI*_*ET*_ and *UI*_*EP*_, which further suggested and confirmed the occurrence of intense upwelling events along the ECPM during the southwest monsoon. A certain condition existed along the northern coast, where the inshore water was relatively warmer than the offshore water, although *UI*_*ET*_ in this region and *UI*_*EP*_ until 5.9°N indicated a favorable upwelling condition. The inshore region of the northern coast is located in the Gulf of Thailand (GoT) where the water was characterized by low salinity water, which primary diluted by several rivers. The fresher water in the GoT was very distinct from the saline SCS water located in the outer GoT, which then caused the formation of salinity front near the entrance of the GoT. Though the region was predominantly characterized by salinity front, however, an attendant SST front was found in the same location [39] and it can be observed at the north of 5°N (Fig 10), which separated the warmer water in the GoT and cooler water in the outer gulf. Since the warmer water was located in the GoT, hence it was no doubt that the riverine outflow water in the GoT was also characterized by warmer continental shelf water and caused warmer SST in the northern coast, dominated the effect of Ekman transport of Ekman pumping that observed to be feeble in the region, insufficient to induce cooler SST. On top of that, a comparable cooler water band was observed to be located away from the northern coast, positioned at the eastern part of the SST front. Previous investigation by Kok et al. [27] suggested this condition was due to the occurrence of northeast flow of offshore surface current in the northern coast that deviated the cooler water from the southern region offshore, forming a cooler water filament that extended northeastward, which then joined the cooler upwelling water in the Vietnamese coast.

Even though the value of *UI*_*SST*_ is generally in good agreement with the computed values of *UI*_*ET*_ and *UI*_*EP*_, it is not entirely precise to conclude that *UI*_*SST*_ during the southwest monsoon was affected only by upwelling. As observed from the SST maps, the cooler water extended from the southern tip of the ECPM, which indicated that there was another source of cooler water transported into the ECPM from the southern region. This transport of cooler water has been firstly documented by Kok et al. [27], where this cooler water was advected from the Java Sea by the northward flowing current in the region. Throughout the southwest monsoon, a continuous change in the SST led to the weakening of thermal fronts and resulted in a constant value of *UI*_*SST*_ of approximately 0.4°C from June to September, although the cooler water patch located along the ECPM experienced a drop in temperature. Nonetheless, in July, although the advection of cooler water from the Java Sea was observed and caused a band of cooler water, but a distinguishable cooler water was also detected along the ECPM, indicated the local upwelling able to further cools the already cool water from the Java Sea in this particular time. While in August and September, the upwelling along the ECPM did not show to further cools the already cooled Java Sea, particularly due to the flood on of Java Sea cooler water was having the similar temperature with the upwelling water or was slightly cooler than the upwelling water.

The two abovementioned conditions, i.e. northward inflow of cooler water and the change in SST suggested that *UI*_*SST*_ was unsuitable for characterizing upwelling in this region; hence, upwelling along the ECPM can only be characterized by using *UI*_*W*_. However, *UI*_*SST*_ in June and July could still be relied upon to characterize due to the absence of cooler water inflow in June and the ability of upwelling to further cools the already cool water from the Java Sea in July.

Wind-induced upwelling is not a rare phenomenon in the coastal ocean. Therefore, it is valuable for us to compare the upwelling between the ECPM with other coastal oceans. In June, upwelling along the ECPM caused the formation of cooler water and resulted in the SST gradient between the inshore and offshore region, similar to the nearby upwelling area in the SCS, such as in the Vietnamese coast [40–43] and northern SCS [44–47]. Nonetheless, the resulted inshore and offshore SST gradient in these areas was usually more than 1°C, larger than those observed along the ECPM. This situation was customarily due to the small vertical temperature difference in the shallow water of the ECPM, as indicated by Kok et al. [27], hence the upwelled water did not had much temperature variation with the surface water. In July, the local upwelling along the ECPM was observed to be able to further cools the already cooled water from the Java Sea where this phenomenon was similar to the eastern boundary currents (EBC) that affiliated with upwelling zones, i.e. the Canary current in the North Atlantic, the Benguela current in the South Atlantic, the California current in the North Pacific, and the Humboldt current in the South Pacific. The EBC carries cooler water equatorward [48,49] and the alongshore prevailing equatorward winds induced extensive upwelling [50–52], further cools the already cool EBC which forming a distinguishable cooler SST along the coast [9,53–57], similar to the ECPM. However, the inshore and offshore SST gradient in the EBC upwelling system was typically larger than that along the ECPM, which it can be exceed 3°C as such as in the Canary upwelling system [9], probably due to the reason as in June.

Examination of both the values of *UI*_*W*_ implied that *UI*_*ET*_ and *UI*_*EP*_ showed an identical trend where the intensity increased in May and peaked in August before declining in September, with the intensity decreasing from south to north along the coastline of the ECPM. Therefore, which wind-driven upwelling mechanism actually serves as the major contributor of upwelling along the ECPM? In order to clarify this, comparison between the wind-driven upwelling mechanisms has been drawn from Tanjung Penyusop until 5.9°N in the northern coast, by integrating Ekman pumping with respect to the distance where the positive wind stress curl existed to determine the vertical transport of Ekman pumping so that it can be compared with Ekman transport [6,7]. In the temporal scale, Ekman pumping was the major contributor of upwelling in any particular month during the southwest monsoon and it contributed to more than half of the total upwelling along the ECPM. While in the spatial scale, Ekman transport was not a major contributor of upwelling in the middle and northern coasts, and it shared half of its contribution with Ekman pumping in the southern coast. Along the ECPM, Ekman transport across all the sections of the coast and Ekman pumping in the middle and northern coasts exhibited a stable pattern; nonetheless, Ekman pumping exhibited an unstable pattern with an existing spike in the southern coast, which was due to the relatively unstable pattern of Ekman pumping that perpendicular to the southern coast, as indicated by Fig 6.

It is crucial to bear in mind that the understanding of Ekman transport and Ekman pumping is based on the classical theory proposed by Ekman [58] in which his original formulation included strong assumptions such as (i) no boundaries, (ii) infinitely deep water, (iii) constant vertical diffusion coefficient, (iv) persistent and steady wind, (v) no geostrophic flow, and (vi) constant Coriolis parameter [59]. In the coastal area, these assumptions are unsatisfied and may cause probable defects to the results obtained from this study. Nonetheless, despite these disagreements, Ekman’s theory still remains to macroscopically describe the near-surface water transport [9]. In addition, it is important to note here that the finest resolution of the ECMWF data is 0.125° × 0.125°, i.e., around 14 km, and consequently, this study does not include the minor characteristics that could lead to Ekman pumping, which will then significantly enhance the coastal upwelling along the ECPM. Hence, finer resolution data or a numerical modeling approach is recommended for future study.

## Conclusions

This study has been conducted to improve and provide new understanding of upwelling along the ECPM, as limited information is available on the dynamics of the system in this region. In the present study, the spatiotemporal variability of upwelling along the ECPM during the southwest monsoon was investigated and characterized in terms of *UI*_*W*_ and *UI*_*SST*_. In addition, the dominant contributor of the wind-induced upwelling mechanism was examined.

In general, throughout the southwest monsoon, *UI*_*ET*_ and *UI*_*EP*_ exhibited a very similar spatiotemporal trend, wherein the value of these two wind-driven upwelling indexes indicated that the upwelling intensity increased in May and peaked in August before declining in September, with an increase in magnitude as it moved southward along the coastline of the ECPM.

In the case of the southwest monsoon, upwelling causes the inshore SST to be cooler than the offshore SST, and it is characterized by an important difference between the inshore and the oceanic SSTs, suggesting the occurrence of upwelling. Nonetheless, it cannot be concluded that the cooler inshore SST was entirely caused by upwelling and it led to *UI*_*SST*_ becoming unsuitable for characterizing upwelling along the ECPM primarily for two reasons. First, it was perceived that the existence of cooler inshore water was under the influence of cooler water advection from the Java Sea, which suggested that the occurrence of cooler inshore water was not entirely caused by upwelling alone. From another point of view, SST along the ECPM exhibited monthly modulation and underwent continuous changes, particularly in the oceanic region. Therefore, upwelling along the ECPM was relatively more suitable and could still be characterized by using *UI*_*ET*_ and *UI*_*EP*_ instead of *UI*_*SST*_, despite some disagreements that occurred with respect to Ekman’s theory.

Although both the Ekman transport and Ekman pumping was said to contribute to the upwelling along the ECPM, however, both wind-induced upwelling mechanisms were found to have a different contribution towards upwelling. Estimation of both wind-induced upwelling mechanisms, i.e., Ekman transport and Ekman pumping, indicated that Ekman pumping was more significant than Ekman transport during all the southwest monsoon months, and this mechanism contributed to about more than half of the total upwelling transport along the entire coastline of the ECPM. On the spatial scale, Ekman transport was less predominant than Ekman pumping in the middle and northern coasts, while Ekman pumping and Ekman transport were equally essential in the southern coast.
